# Complete chloroplast genome sequence of the carnivorous herb *Pinguicula alpina* (Lentibulariaceae)

**DOI:** 10.1080/23802359.2022.2086075

**Published:** 2022-06-30

**Authors:** Jianfang Li, Zhan-Lin Liu

**Affiliations:** Key Laboratory of Resource Biology and Biotechnology in Western China (Ministry of Education), College of Life Sciences, Northwest University, Xi’an, China

**Keywords:** *Pinguicula alpina*, plastome, phylogeny

## Abstract

We determined the complete chloroplast genome of *Pinguicula alpina*, a carnivorous plant using high-throughput sequencing technology. The deduced plastome is a closed circular molecule of 147,479 bp with a quadripartite structure, including a large single-copy region (LSC) of 81,937 bp, a small single-copy region (SSC) of 13,180 bp, and a pair of inverted repeat regions (IRs) of 26,181 bp. It contains 131 genes, comprising 82 protein-coding genes, 37 tRNA genes, and 8 rRNA genes. The GC content of the plastome, LSC, SSC, and IR regions are 38.1%, 36.1%, 31.4% and 43.0%, respectively. Phylogenetic analysis revealed that *P. alpina* was related to *P. ehlersiae.*

*Pinguicula* Linnaeus 1753, a genus of carnivorous plants in Lentibulariaceae family, is extensively distributed throughout Eurasia and America. The genus comprises approximately 100 species with diverse morphological traits. Infrageneric relationships have not been clarified, although chloroplast and nuclear fragments have been used to explore the phylogenetic issues (Cieslak et al. 2005; Degtjareva et al. 2006). *Pinguicula alpina* Linnaeus 1753, a perennial herb that prefers wet soil in open locations, can be found at high altitudes and latitudes in Eurasia (Wu et al. 2011). Despite its wide distribution, its habitat is fragile and is threatened by global climate change and agricultural development. Carnivorous plants depend deeply on insects for pollination and nutrition. The changing climate affects the behavior of insects, which further affects the reproduction and prey capture in the carnivorous plants (Primer et al. 2018). Genetic information is essential for conservation management and mapping evolutionary trajectories. In this study, we determined the complete chloroplast genome using high-throughput sequencing technology to provide useful resources for phylogenetic analysis of *Pinguicula* and population conservation studies in *P. alpina*.

Fresh leaves were collected from an individual in the Qinling Mountains, China (N33.99° E107.75°). The specimen has been deposited at the herbarium of Northwest University (contact person: Zhan-Lin Liu, liuzl@nwu.edu.cn) with voucher number 2017LIU023. Specific permits and ethical approval were not required for specimen collection. This study complied with the relevant laws of China. Total DNA isolated by modified CTAB method was applied to 250-bp paired-end library construction. Genome sequencing was performed using an Illumina HiSeq2000 device (Novogene Co. Ltd). Genome assembly was performed by GetOrganelle v1.73 (Jin et al. 2020) and annotated using Geneious v9.0 (Kearse et al. 2012) with *P. ehlersiae* (NC_023463) as the reference.

The plastome size of *P. alpina* (MT740255) is 147,479 bp, with a large single-copy region (LSC) of 81,937 bp, a small single-copy region (SSC) of 13,180 bp, and a pair of inverted repeat regions (IRs) of 26,181 bp. It contains 131 genes, including 82 protein-coding genes, 37 tRNA genes and 8 rRNA genes. Eighteen genes are duplicated in the IRs, including seven protein-coding genes, seven tRNA genes, and four rRNA genes. Fifteen genes harbor a single intron and three genes (*ycf3*, *rps12*, and *clpP*) have two introns. The overall GC content of the plastome is 38.1%. The GC values of the LSC, SSC and IR regions are 36.1%, 31.4%, and 43.0%, respectively.

To elucidate the phylogenetic position of *P. alpina* in Lentibulariaceae, 14 chloroplast genomes were used to construct the maximum likelihood tree using RaxmlGUI v2.0 (Edler et al. 2021) with 1000 rapid bootstrap replicates, and *Salvia japonica* (NC_035233) and *Lavandula angustifolia* (NC_029370) as the outgroups. A general time-reversible model was selected for the best substitution of DNA using ModelTest-NG (Darriba et al. 2020). The phylogenetic tree demonstrated that *P. alpina* was grouped with *P. ehlersiae* (Figure 1). This plastome information could be utilized to evaluate the phylogenetic relationships and mapping evolutionary patterns of Lentibulariaceae taxa.

**Figure 1.**
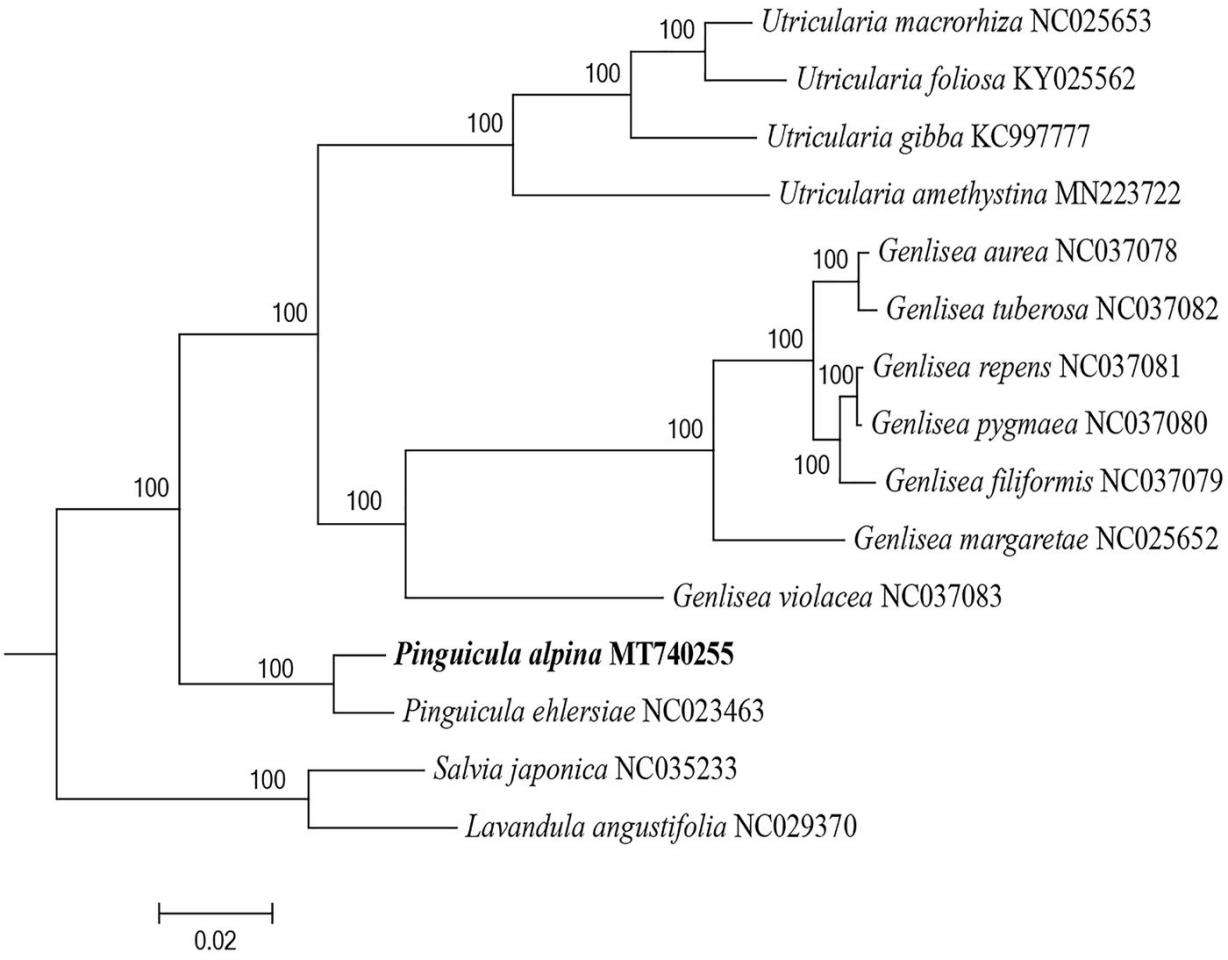
The phylogenetic tree constructed by the complete chloroplast genome sequences in Lentibulariaceae. The bootstrap support values based on 1000 replicates were 100% in all branches.

## Ethics statement

Ethical approval is not applicable for the study.

## Author contributions

Li J analyzed the data and wrote the drafting of the paper. Liu Z-L designed the experiments, revised and approved the final version of the manuscript. All authors agree to be accountable for all aspects of the work.

## Data Availability

The data that support the findings of this study are openly available in the National Center for Biotechnology Information (NCBI) at https://www.ncbi.nlm.nih.gov with the reference number MT740255. The associated BioProject, SRA and Bio-Sample number are PRJNA799402, SRX13861202, and SAMN25164382, respectively.
